# Heme Oxygenase-1 Inhibits HLA Class I Antibody-Dependent Endothelial Cell Activation

**DOI:** 10.1371/journal.pone.0145306

**Published:** 2015-12-21

**Authors:** Eva Zilian, Hendry Saragih, Vijith Vijayan, Oliver Hiller, Constanca Figueiredo, Abid Aljabri, Rainer Blasczyk, Gregor Theilmeier, Jan Ulrich Becker, Jan Larmann, Stephan Immenschuh

**Affiliations:** 1 Institute for Transfusion Medicine, Hannover Medical School, Hannover, Germany; 2 Faculty of Biology, Gadjah Mada University, Yogyakarta, Indonesia; 3 Department of Anesthesiology and Intensive Care Medicine, Hannover Medical School, Hannover, Germany; 4 Institute of Pathology, University Hospital of Cologne, Cologne, Germany; Faculty of Biochemistry, Biophysics and Biotechnology, Jagiellonian University, POLAND

## Abstract

Antibody-mediated rejection (AMR) is a key limiting factor for long-term graft survival in solid organ transplantation. Human leukocyte antigen (HLA) class I (HLA I) antibodies (Abs) play a major role in the pathogenesis of AMR via their interactions with HLA molecules on vascular endothelial cells (ECs). The antioxidant enzyme heme oxygenase (HO)-1 has anti-inflammatory functions in the endothelium. As complement-independent effects of HLA I Abs can activate ECs, it was the goal of the current study to investigate the role of HO-1 on activation of human ECs by HLA I Abs. In cell cultures of various primary human macro- and microvascular ECs treatment with monoclonal pan- and allele-specific HLA I Abs up-regulated the expression of inducible proinflammatory adhesion molecules and chemokines (vascular cell adhesion molecule-1 [VCAM-1], intercellular cell adhesion molecule-1 [ICAM-1], interleukin-8 [IL-8] and monocyte chemotactic protein 1 [MCP-1]). Pharmacological induction of HO-1 with cobalt-protoporphyrin IX reduced, whereas inhibition of HO-1 with either zinc-protoporphyrin IX or siRNA-mediated knockdown increased HLA I Ab-dependent up-regulation of VCAM-1. Treatment with two carbon monoxide (CO)-releasing molecules, which liberate the gaseous HO product CO, blocked HLA I Ab-dependent EC activation. Finally, in an *in vitro* adhesion assay exposure of ECs to HLA I Abs led to increased monocyte binding, which was counteracted by up-regulation of HO-1. In conclusion, HLA I Ab-dependent EC activation is modulated by endothelial HO-1 and targeted induction of this enzyme may be a novel therapeutic approach for the treatment of AMR in solid organ transplantation.

## Introduction

Antibody (Ab)-mediated rejection (AMR) is a major limiting factor for long-term graft survival after kidney and heart transplantation [1–4]. The endothelium of allografts plays a key role in the pathogenesis of AMR [5, 6], because it is targeted by donor-specific Abs (DSAs), which are directed against human leukocyte antigen (HLA) and/or non-HLA molecules [7, 8]. It is well established that HLA Abs can cause EC injury by complement fixation [7, 9], but more recently, complement-independent effects of HLA Abs have also been implicated in AMR [10–13]). Although the mechanisms of how these Abs mediate EC damage independent of complement fixation are not completely understood, HLA class I (HLA I) Abs have been shown to directly cause activation of ECs [5, 6]. EC activation is critically involved in the pathogenesis of acute and chronic inflammation [14]. Moreover, it is characterized by alterations of intracellular endothelial signaling, which up-regulates expression of inducible adhesion molecules and chemokines [15], and in turn modulates coordinated recruitment of leukocytes to the site of inflammation [16, 17].

Current therapeutic regimens for AMR such as plasmapheresis and treatment with CD20 Abs (rituximab) are primarily intended to reduce levels of circulating pathogenic DSAs [4, 18, 19]. The clinical success rate of these therapies, however, is limited, and alternative treatment strategies are urgently needed. The antioxidant enzyme heme oxygenase (HO)-1, which is the inducible isoform of catalytic heme degradation [20], has previously been shown to have protective effects in the endothelium [21, 22]. Moreover, overexpression of HO-1 has been shown to inhibit up-regulation of proinflammatory adhesion molecules in tumor necrosis factor (TNF)-α-activated ECs [23] and to have anti-inflammatory therapeutic potential in various cardiovascular disorders [24–27]. In transplantation settings, survival of cardiac xenografts has been linked to endothelial HO-1 in a mouse-to-rat heart transplantation model [28] and genetic transfer of HO-1 into blood vessel walls has been shown to protect against allogeneic rejection of aortic vascular transplants [29]. Moreover, HO-1 has been demonstrated to have beneficial effects against complement-mediated damage of HLA Abs [30].

In the current study, we hypothesized that HO-1 may specifically modulate HLA I Ab-dependent activation of ECs. It is demonstrated that HLA I Abs up-regulate inducible adhesion molecules and chemokines in human ECs and cause increased endothelial adhesion of monocytes. Both HLA I Ab-dependent effects in ECs are modulated by specific regulation of HO-1.

## Materials and Methods

### Abs and chemicals

The murine monoclonal antibodies (MoAbs) W6/32 and G46-2.6, both of which are directed against distinct monomorphic HLA I epitopes, were from either ATCC (Manassas, VA, USA)(W6/32) or BD Bioscience (San Jose, CA, USA)(G46.2.6) and were prepared as previously described [31]. The murine isotype control MoAb (MCA929EL) was from AbD Serotec (Oxford, UK), human HLA-A2 (clone BB7.2) and microglobulin-2 2b-57 as IgG2b isotype control were from Biolegend (London, UK). Polyclonal rabbit anti-human VCAM-1 and anti-human glyceraldehyde dehydrogenase (GAPDH) for Western blot analyses were from Santa Cruz Biotechnology (Santa Cruz, CA, USA), polyclonal rabbit anti-human HO-1 from Enzo Life Sciences (Lörrach, Germany). Secondary HRP-conjugated goat anti-rabbit IgG was from BioRad (Hercules, CA, USA), murine monoclonal VCAM-1 (51-10C9) for blocking leukocyte binding to VCAM-1 on ECs from BD Biosciences (Heidelberg, Germany) and isotype mouse IgG1κ control Ab (MOPC-21) from Biolegend (London, UK). Phosphatidylinositol 3-kinase (PI3K)/Akt inhibitors wortmannin and LY294002, NF-κB inhibitors MG132 and Bay 11–7082 or extracellular-regulated kinase (ERK) inhibitors UO126 and PD98059 were from Merck Biosciences (La Jolla, CA, USA). Cobalt-protoporphyrin (CoPPIX) and zinc-protoporphyrin (ZnPPIX) were from Frontiers Scientific (Logan, Utah, USA), carbon monoxide (CO)-releasing molecules (CORMs), CORM-2 and -3, were from Sigma-Aldrich (Steinheim, Germany) and TNF-α from PeproTech (Rocky Hill, NJ, USA).

### Cell cultures and treatment of EC cultures with Abs and chemicals

Human umbilical vein endothelial cells (HUVECs) and human dermal microvascular endothelial cells (HDMVECs) were from PromoCell (Heidelberg, Germany) and human aortic endothelial cells (HAECs) were from Lonza (Cologne, Germany). For studies with HLA-typed cells, HUVECs with different HLA I genotypes (Donor 1 (Lot. # 696527): A*02, A*30, B*15, B*44; donor 2 (Lot. #1022301.1): A*02, A*26, B*35, B*39; donor 3 (Lot. # 1050901): A*01, A*11, B*08, B*39) were applied. Cells were used in passages 4 to 7 and were cultured in 1% gelatine-coated flasks in EC Growth Medium 2 (PromoCell) and 5% (vol/vol) heat-inactivated fetal calf serum (FCS)(Lonza). Cells were maintained until confluency at 37°C in a controlled environment of 100% humidity and 5% CO_2_. For HLA I Ab simulation experiments ECs were cultured in 12-well flat bottom dishes with 2 ml EC Growth Medium 2 plus 5% FCS until confluency. After an overnight starving period in medium containing 2% FCS, cells were stimulated with 10 μg/ml of HLA I Abs or isotype control Abs unless otherwise indicated. For inhibitor studies, HUVECs were treated with PI3K/Akt inhibitors wortmannin (1 μM) and LY294002 (4 μM), the NF-κB inhibitors MG132 (100 nM) and Bay 11–7082 (10 μM), the ERK inhibitors UO126 (20 μM) and PD98059 (10 μM), the HO modulators CoPPIX and ZnPPIX at a final concentration of 5 μM or CORM-2 (25 μM) and CORM-3 (75 μM) for 30 min prior to treatment with HLA I Abs. Inactivated CORMs (iCORMs), iCORM-2 and -3, which have released CO and serve as a control, were prepared as previously described [32].

### Analysis of mRNA expression

Expression of mRNA was determined as previously described [33]. In detail, for RNA extraction the RNeasy mini kit (Qiagen, Hilden, Germany), for cDNA synthesis with the High Capacity cDNA Reverse Transcription Kit (Applied Biosystems, Darmstadt, Germany) were applied. Inventoried primer mixes for quantification of mRNA levels of VCAM-1, ICAM-1, MCP-1, IL-8, HO-1 and cyclooxygenase (Cox)-2 were from Applied Biosystems. Amplification was performed with TaqMan Gene Expression Master Mix on a StepOnePlus™ Real-Time PCR System (Applied Biosystems). Thermal cycling was performed at 95°C for 10 min followed by 40 cycles at 95°C for 15 s and 60°C for one min. GAPDH was used as a control for normalization of cDNA levels and the *ΔΔ*CT method was applied for determining mRNA levels semi-quantitatively according to the manufacturer's protocol.

### Western blot analysis

Western blotting was performed with primary antibodies against VCAM-1 (1:1,000) or HO-1 (1:1,000) and secondary horse radish-conjugated goat anti-rabbit Ab (1:10,000), as previously described [34]. Signals were visualized by enhanced chemiluminescence (Roth GmbH, Karlsruhe, Germany) and quantified with a Fluorchem (Alpha Innotec, San Leandro, CA, USA). Images were processed using Corel Draw Graphic Suite X5 Software (Corel Corporation, Ottawa, Canada).

### Flow cytometry

The surface expression of VCAM-1 in HUVECs after treatment with W6/32 for 24 h was analysed by flow cytometry. Briefly, 1x10^6^ cells were stained with phycoerythrin-conjugated monoclonal VCAM-1 antibody (BD Pharmingen^TM^) or the isotype control (BD pharmingen^TM^) according to the manufacturer’s protocol. The analyses were performed using a FACS Canto II flow cytometer (BD Biosciences) and FACSDiva Software (V6.1.2). At least 20,000 events were used for the analyses and the average mean fluorescence intensity (MFI) from 3 independent experiments was calculated.

### Knockdown of HO-1 in HUVECs

HUVECs were cultured in 12-well flat bottom dishes containing 1 ml EC Growth Medium 2 supplemented with 5% FCS and transfection was carried out with Oligofectamine™ (Life Technologies, Darmstadt, Germany) and Stealth™ RNAi (or siRNA) for HO-1 (Thermo Scientific). 150 pmol of Stealth™ RNAi were diluted in 77.5 μl of Opti-MEM I Reduced Serum Medium (Life Technologies) and were incubated for 15 min. 4 μl oligofectamine™ were mixed with 11 μl of Opti-MEM I Reduced Serum Medium for 5 min at room temperature. Both dilutions were combined and incubated for 15 min at room temperature. After removal of growth medium, cells were washed with pre-warmed Opti-MEM I medium, and 400 μl Opti-MEM I medium plus 100 μl of Stealth RNAi-Oligofectamine complexes was added. After 4 h incubation 250 μl of EC Growth Medium 2 containing 15% FCS was added to the cells without removing the transfection mixture.

### Leukocyte adhesion assay

This assay was performed as previously described [35]. In detail, HUVECs were cultured in 6-well cell culture plates to confluency before incubation with W6/32 for 18 h. For blocking of VCAM-1, cells were co-incubated with 10 μg/ ml mouse anti-human VCAM-1 Ab or the appropriate control Ab. Adhesion of the monocyte cell line THP-1 (ATCC, Manassas, VA) to HUVECs was determined as previously described with Cell tracker green (Life Technologies)[35]. Adhering macrophages were counted by fluorescence microscopy in 15 preselected high-power fields by a blinded investigator. Images were acquired at room temperature using an Olympus IX81 microscope (Olympus, Hamburg, Germany). A QImaging Retiga EXi camera (QImaging, Surrey, BC, Canada) and QCapture Pro Software version 6.0.0.412 (QImaging) were used to capture and analyze immunofluorescence images.

### Statistical analysis

Quantitative data from Western blot analyses, real-time RT-PCR experiments and adhesion assays were analysed by two-tailed Student *t* test and are presented as mean ± SEM from at least three independent experiments. A p-value p≤0.05 was considered as statistically significant in all analyses.

## Results

### HLA I Abs up-regulate the expression of inducible adhesion molecules and chemokines in human ECs

**Fig 1:**
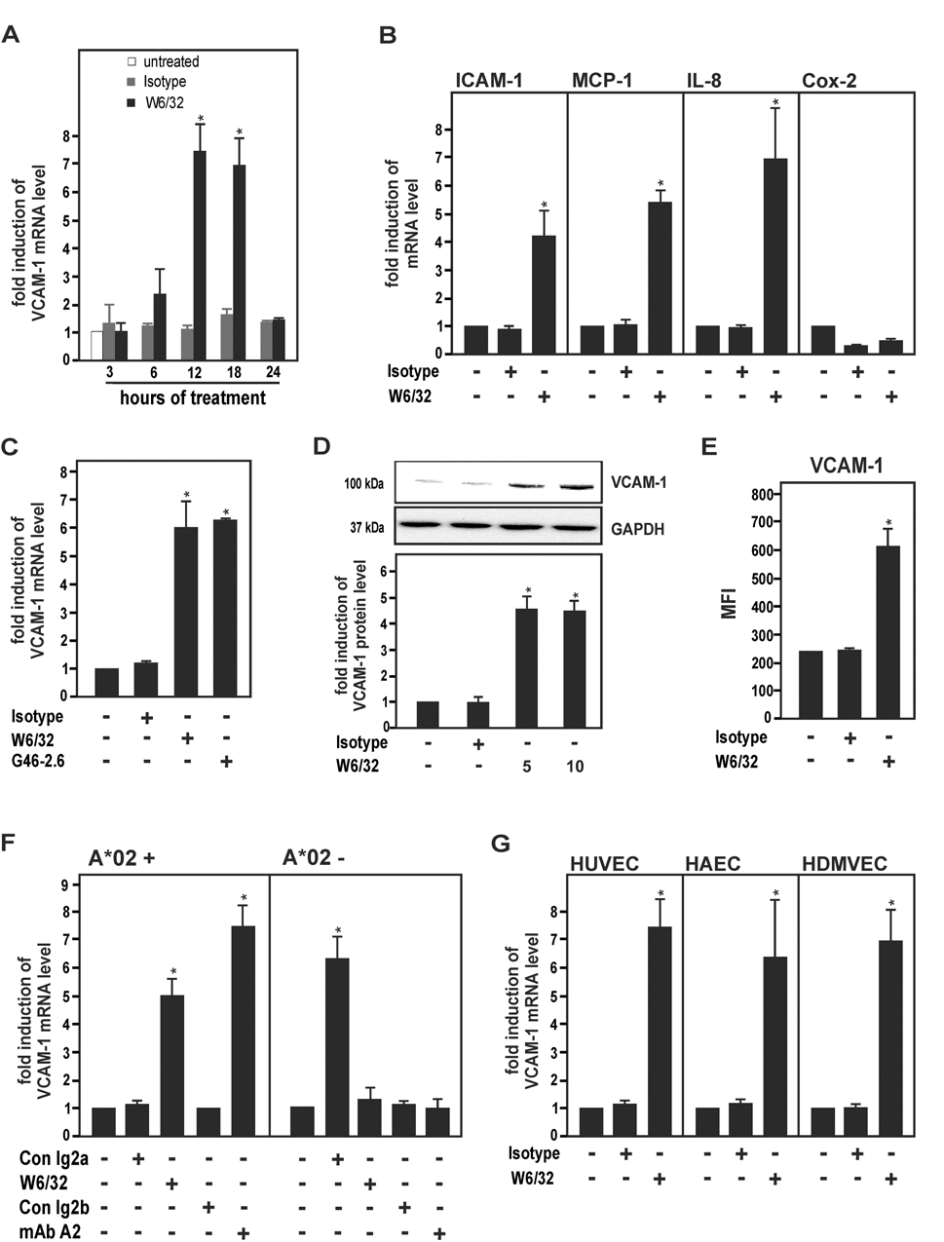
Up-regulation of VCAM-1 and other proinflammatory inducible genes by HLA I Abs in cell cultures of human ECs. HUVECs **(A-E)**, HAECs and HDMVECs **(F)** were cultured as described under Materials and Methods. **(A)** HUVECs were treated with MoAb W6/32 or isotype control (10 μg/ml) for the indicated times. Cells were lysed and subjected to RT-PCR analysis. The fold induction of VCAM-1 mRNA levels is shown. **(B)** HUVECs were treated with W6/32 or isotype control for 18 h. Cells were lysed and subjected to RT-PCR analysis for ICAM-1, MCP-1, IL-8 and Cox-2. The fold induction of mRNA levels is shown. **(C)** HUVECs were treated with MoAbs W6/32 and G46-2.6 or isotype control (10 μg/ml) for 18 h. Cells were lysed and subjected to RT-PCR analysis. The fold induction of VCAM-1 mRNA levels is shown. **(D)** HUVECs were treated with W6/32 (5 and 10 μg/ml) or isotype control for 18 h, as indicated. Cells were subjected to Western blot analysis and probed with VCAM-1 and GAPDH Abs. The fold induction of protein levels is indicated and a representative of three independent experiments is shown. **(E)** HUVECs were treated with W6/32 or isotype control (10 μg/ml) for 24 h. The surface expression of VCAM-1 was analysed by flow cytometry. The average MFI calculated from three independent experiments is shown. **(F)** HLA class I-genotyped HUVECs (HLA-A*02^+^ or HLA-A*02^-^) were treated with W6/32 and an allele-specific MoAb against HLA-A2 (clone BB7.2) or isotype controls for 18 h. Cells were lysed and subjected to RT-PCR analysis. The fold induction of VCAM-1 mRNA levels is shown. **(G)** HUVECs, HAECs and HDMVECs were treated with W6/32 or isotype control Ab (10 μg/ml) for 18 h. Cells were lysed and subjected to RT-PCR analysis. The fold induction of mRNA levels is indicated. All data are means ± SEM from three independent experiments. * p≤ 0.05, significant differences treatment versus control. MFI, mean fluorescence intensity.

The expression of inducible adhesion molecules (VCAM-1, ICAM-1) and chemokines (MCP-1, IL-8) in response to the pan-HLA I MoAb W6/32 was determined in cell cultures of HUVECs [36–38]. W6/32 markedly increased mRNA levels of VCAM-1 in a time-dependent manner with a maximum after 12 h (Fig 1A). Moreover, expression of ICAM-1, MCP-1 and IL-8 was up-regulated in response to W6/32. In contrast, the inducible proinflammatory enzyme Cox-2, which has previously been shown to be induced in TNF-α-activated ECs [39], was down-regulated by W6/32 in HUVECs (Fig 1B). In the following, VCAM-1 was applied as a surrogate marker for HLA I Ab-dependent EC activation. Treatment of HUVECs with a second independent pan-HLA I MoAb, G46-2.6, also up-regulated VCAM-1 expression (Fig 1C). Cellular protein and surface expression levels of VCAM-1 were up-regulated in HUVECs after treatment with W6/32 as determined by Western blot and flow cytometry analysis (Fig 1D and 1E).

To confirm the above findings for an allele-specific HLA I Ab, cell cultures of HLA-genotyped HUVECs were treated with the MoAb BB7.2, which is directed against a specific HLA-A2 epitope. MoAb BB7.2 up-regulated VCAM-1 mRNA expression in HUVECs from two donors with genotype HLA-A*02^+^ (donor 1, A*02, A*30; donor 2, A*02, A*26)(Fig 1F), but not in HUVECs from a donor with HLA-A genotype A*02^-^ (donor 3, A*01, A*11). To determine whether HLA I Ab-mediated up-regulation of VCAM-1 would also apply to adult ECs from micro- and macrovascular blood vessels, we used cell cultures of HAECs and HDMVECs. Similar to the gene regulatory pattern in HUVECs, VCAM-1 mRNA levels were increased by W6/32 in HAECs and HDMVECs (Fig 1G). Taken together, the data indicate that HLA I Abs activate human ECs of different origins and up-regulate the expression of inducible adhesion molecules and chemokines in these cells.

### HLA I Ab-dependent up-regulation of VCAM-1 is mediated via activation of PI3K/Akt and NF-κB

**Fig 2:**
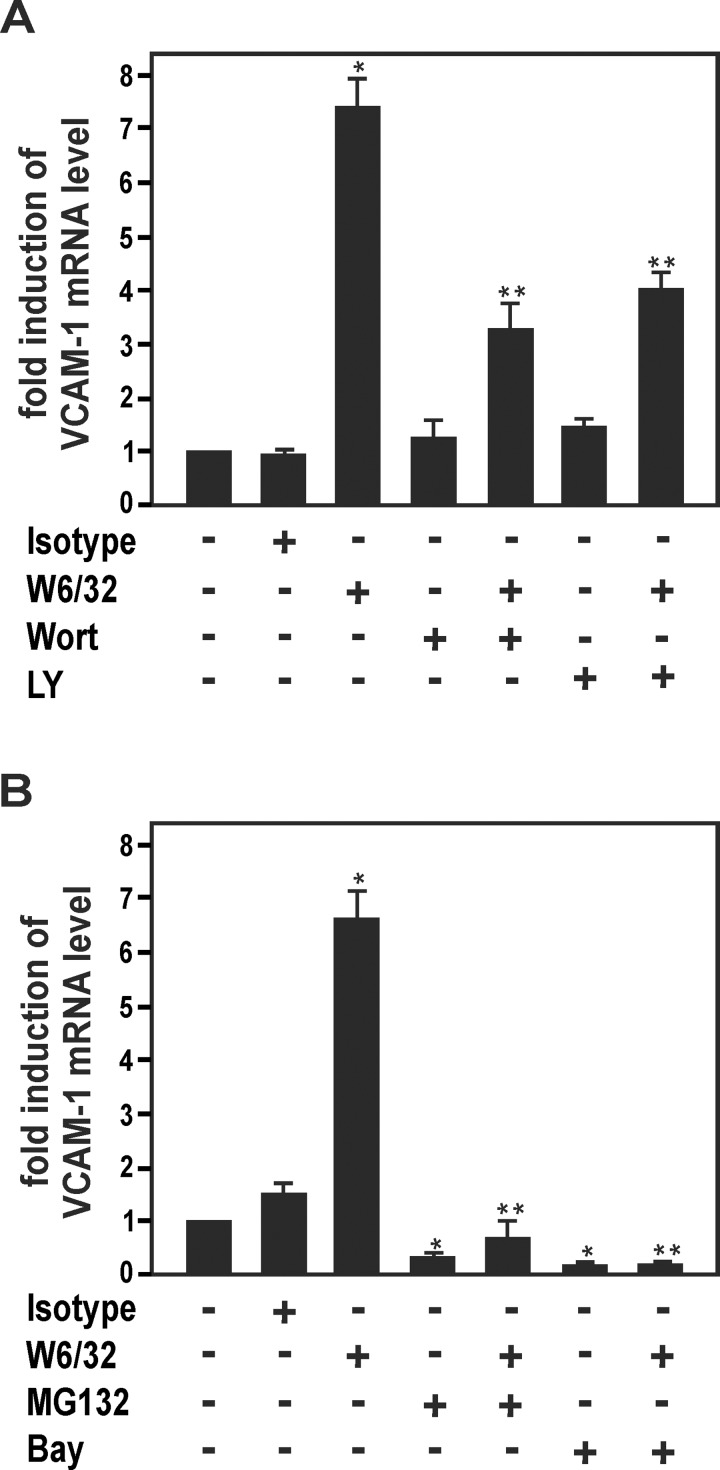
Signaling cascades of HLA I Ab-dependent VCAM-1 up-regulation in HUVECs. **(A, B)** HUVECs were incubated with HLA I Ab W6/32 or isotype control (10 μg/ml) for 18 h after pre-treatment for 30 min with, **(A)** the PI3K/Akt pathway inhibitors wortmannin (1 μM) and LY294002 (4 μM), or with, **(B)** the NF-κB pathway inhibitors MG132 (100 nM) and Bay 11–7085 (10 μM). Cells were lysed and subjected to RT-PCR analysis. The fold induction of mRNA levels is indicated. Data are mean ± SEM from three independent experiments. * p≤ 0.05, significant differences treatment versus control; ** p≤ 0.05, W6/32 versus W6/32 plus inhibitor. Wort, wortmannin; LY, LY294002; Bay, Bay 11–7085.

Ligation of HLA I Abs to ECs has been shown to activate various intracellular signaling cascades such as PI3K/Akt, ERK and NF-κB [5]. To investigate the potential role of these regulatory pathways in HLA I Ab-dependent up-regulation of VCAM-1, HUVECs were pretreated with various specific pharmacological inhibitors. The PI3K/Akt inhibitors wortmannin and LY294002 markedly decreased up-regulation of VCAM-1 mRNA levels in HUVECs by W6/32 (Fig 2A). In contrast, ERK1/2 inhibitiors UO126 or PD09059 had no effect on VCAM-1 up-regulation by W6/32 (data not shown). In addition, W6/32-dependent induction of VCAM-1 was blocked by two pharmacological inhibitors of the NF-κB pathway, MG132 and Bay 11–7082 (Fig 2B). Blocking of PI3K/Akt and NF-κB also inhibited W6/32-dependent induction of ICAM-1, MCP-1 and IL-8 (data not shown). Taken together, the PI3K/Akt and NF-κB cascades are involved in HLA I Ab-mediated up-regulation of inducible adhesion molecules and chemokines in human ECs.

### Modulation of HO-1 affects HLA I Ab-dependent induction of VCAM-1 in HUVECs

**Fig 3:**
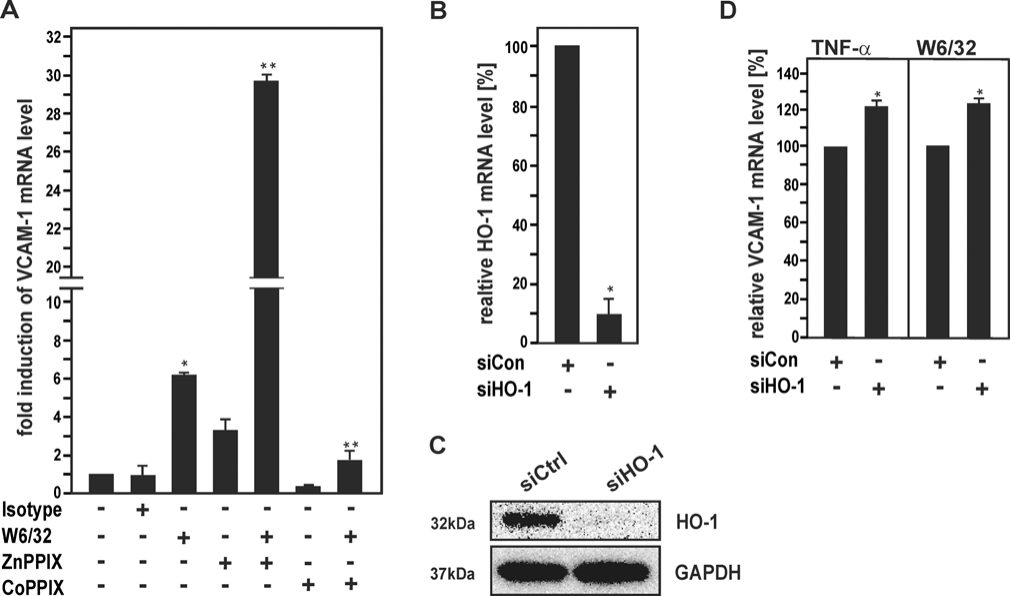
Pharmacological inhibition and siRNA-mediated knockdown of HO-1 reduce HLA I Ab-induced VCAM-1 expression in HUVECs. **(A)** HUVECs were incubated with HLA I Ab W6/32 alone (10 μg/ml) and for 18 h in the presence of CoPPIX (5 μM) or ZnPPIX (5 μM), as indicated. Cells were lysed and subjected to RT-PCR analysis. The fold induction of VCAM-1 mRNA levels is shown. **(B-D)** HUVECs were transfected with siRNA for HO-1 or scrambled control siRNA. **(B)** mRNA expression was determined by RT-PCR analysis and relative levels of HO-1 mRNA are shown. **(C)** Protein expression was determined by Western blot analysis and probed sequentially with Abs against HO-1 and GAPDH. A representative of three independent experiments is shown. **(D)** Transfected HUVECs were treated with TNFα (15 ng/ml) or W6/32 for 18 h. Cells were lysed and subjected to RT-PCR analysis. The fold induction of mRNA levels is indicated. Bar graphs represent mean ± SEM from three independent experiments. * p≤ 0.05, significant differences treatment versus control; ** p≤ 0.05, W6/32 versus W6/32 plus CoPPIX/ ZnPPIX. Con, control.

**Fig 4:**
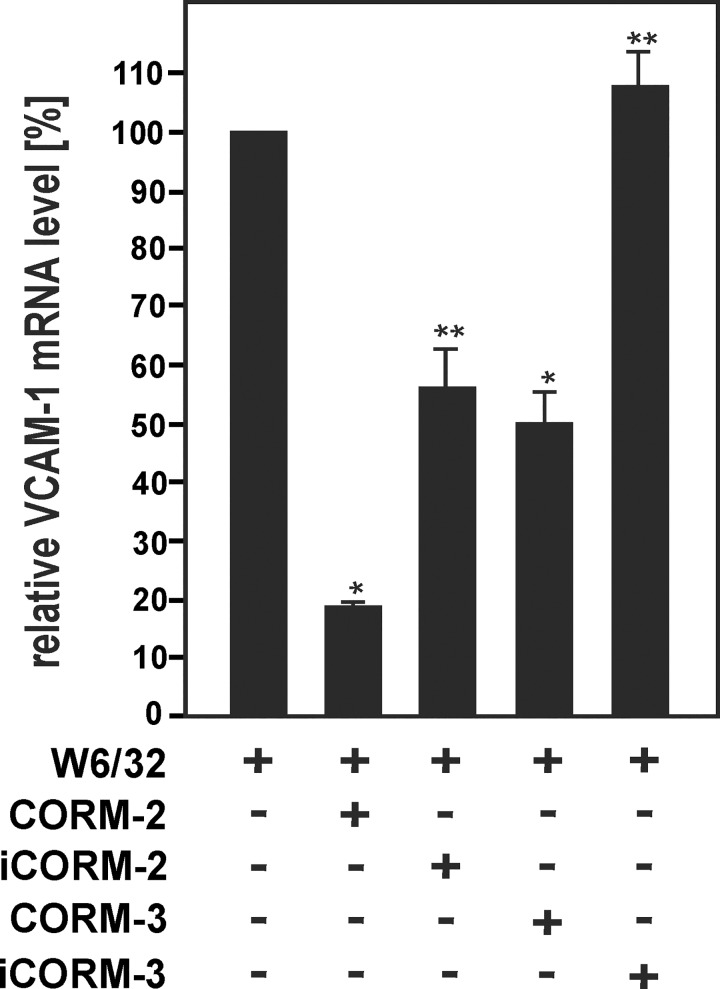
CORMs decrease HLA class I Ab-induced VCAM-1 expression in HUVECs. Confluent HUVECs were incubated with W6/32 or isotype control (10 μg/ml) for 18 h in the presence or absence of CORM-2 (25 μM), iCORM-2 (25 μM), CORM-3 (75 μM) or iCORM-3 (75 μM), as indicated. Cells were lysed and subjected to RT-PCR analysis. The fold induction of mRNA levels is indicated. Data are represented as mean ± SEM from three independent experiments. * p≤ 0.05, significant differences CORM-2/3 versus W6/32; ** p≤ 0.05, iCORM-2/3 versus CORM2/3.

HO-1 has anti-inflammatory effects via its EC-specific functions [24–26] and has previously been shown to reduce up-regulation of several adhesion molecules in TNF-α-activated cell cultures of bovine ECs via inhibition of NF-κB [23, 40]. To investigate whether HO-1 might also affect HLA I Ab-mediated activation of human ECs, HUVECs were treated with W6/32 together with the pharmacological HO inhibitor ZnPPIX or the HO-1 inducer CoPPIX, respectively. Inhibition of HO-1 enzyme activity by ZnPPIX markedly enhanced VCAM-1 mRNA induction after stimulation of HUVECs with W6/32 (Fig 3A). By contrast, CoPPIX-dependent up-regulation of HO-1 reduced VCAM-1 mRNA levels in W6/32-treated HUVECs. To confirm the role of HO-1 in HLA I Ab-mediated VCAM-1 induction, HO-1 was also down-regulated in HUVECs by knockdown with siRNA against HO-1 (Fig 3B and 3C). Treatment of HO-1-depleted HUVECs with either TNF-α or MoAb W6/32 enhanced relative VCAM-1 mRNA levels as compared to control cells, respectively (Fig 3D). The HO product CO has previously been shown to have major anti-inflammatory effects in the endothelium [41, 42]. To study the effects of CO on HLA I Ab-dependent EC activation, HUVECs were exposed to two CORMs, CORM-2 and CORM-3 [42], prior to stimulation with W6/32. Incubation with CORMs markedly decreased up-regulation of VCAM-1 mRNA levels in W6/32-treated HUVECs (Fig 4). Taken together, the data suggest that HO-1 and its product CO inhibit the up-regulation of VCAM-1 in HLA I antibody-activated ECs.

### HO-1 modulates increased HLA I Ab-mediated adhesion of THP-1 monocytes to HUVECs

**Fig 5:**
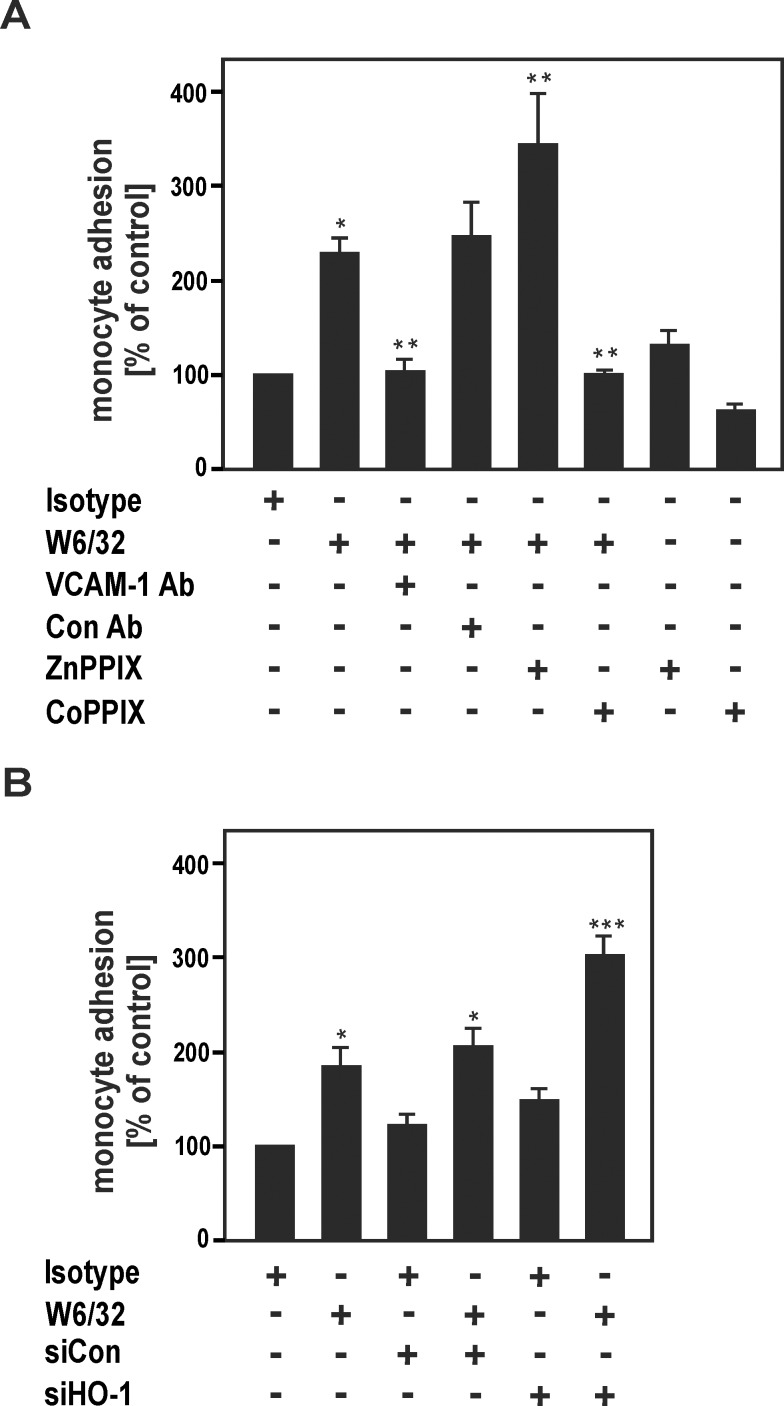
HO-1 modulates HLA I Ab-dependent up-regulation of THP-1 monocyte adhesion to HUVECs. (A) Confluent HUVECs were cultured for 24 h in the presence of MoAb W6/32 or isotype control Ab (10 μg/ml) in the presence or absence of a blocking antibody against VCAM-1, a control Ab (Con Ab), ZnPPIX (5 μM) and CoPPIX (5 μM), as indicated. (B) HUVECs were transfected with siRNA for HO-1 or scrambled control siRNA, as indicated. After the various treatments in (A) and (B) cell cultures were incubated with Cell Tracker green-labeled THP-1 monocytes for 30 min. After 5 washing steps firmly adherent monocytes were quantified with fluorescence microscopy in 15 preselected high-power fields by a blinded investigator. The monocyte adhesion in % of control is indicated. Data are mean ± SEM from three independent experiments. * p≤ 0.05, significant differences treatment versus control; ** p≤ 0.05, W6/32 versus W6/32 plus anti-VCAM-1/ CoPPIX/ ZnPPIX; *** p≤ 0.05, siCon plus W6/32 versus siHO-1 plus W6/32.

HLA I Abs have been shown to enhance adhesion of mononuclear cells to the endothelium in various experimental *in vivo* and *in vitro* models [43, 44]. To address the question whether HLA I Ab-mediated adhesion of mononuclear cells to HUVECs might be modulated by HO-1, we performed *in vitro* leukocyte adhesion assays. HLA I Abs enhanced adhesion of THP-1 monocytes to HUVECs, which was inhibited by a VCAM-1 blocking Ab, but not by an unspecific control Ab. These findings indicate that up-regulation of VCAM-1 by W6/32 is functionally involved in recruitment of monocytes to ECs. To determine the specific effects of HO-1 modulation on monocyte adhesion to ECs, HUVECs were treated with W6/32 in the presence or absence of ZnPPIX and CoPPIX. Whereas ZnPPIX increased, CoPPIX reduced W6/32-dependent adhesion of monocytes to HUVECs (Fig 5A). Similarly, knockdown of HO-1 led to a marked up-regulation of monocyte adhesion to ECs (Fig 5B). The data suggest that HLA I Ab-dependent increase of monocyte adhesion to HUVECs is inhibited by HO-1 up-regulation in human ECs.

## Discussion

AMR plays a key role in graft rejection after solid organ transplantation and is a major clinical challenge due to the lack of feasible therapeutic regimens [1–4]. In addition to well-known complement-dependent effects of DSAs, complement-independent effects of such Abs have recently been implicated in the pathogenesis of AMR [13] via activation of ECs [5, 6]. In the current study, we investigated, whether the anti-inflammatory enzyme HO-1 can modulate HLA I Ab-dependent EC activation. The major findings of the current study are, that 1) HLA I Abs up-regulate inducible adhesion molecules and chemokines in cell cultures of primary human ECs via activation of PI3K/Akt and NF-κB; 2) HO-1 specifically modulates HLA I Ab-dependent expression of these inducible proinflammatory genes; 3) HO-1 counteracts the increased HLA I Ab-dependent adhesion of monocytes to ECs.

### Activation of human ECs by HLA I Abs

The current report shows that treatment with two pan-HLA I MoAbs (W6/32, G46.6) and an allele-specific HLA MoAb (BB7.2) up-regulates the gene expression of inducible proinflammatory adhesion molecules and chemokines in cell cultures of various primary human ECs (Fig 1). These findings agree with earlier reports, in which it has been shown that HLA I Abs activate human ECs via complement-independent effects ([43, 45–48]; for reviews see [5, 44, 49]). As an example, VCAM-1 has been shown to be up-regulated by HLA Abs eluted from acutely rejected renal allografts in cultured HUVECs [37]. Moreover, it has been demonstrated that HLA I Abs up-regulate vascular endothelial growth factor expression in cell cultures of HUVECs [50] or VCAM-1 and ICAM-1 in the human microvascular EC line HMEC-1 [36], respectively. Conflictingly, others have reported that VCAM-1 might not be regulated in response to HLA I Abs [48, 51]. These contradictory observations could be explained by cell type-specific differences of various ECs and/or variations of cell culture conditions in these studies. In accordance with earlier findings by others [52], we also observed that the PI3K/Akt cascade is involved HLA I Ab-dependent up-regulation of inducible adhesion molecules and chemokines (Fig 2). Interestingly, in contrast to a previous report [36], ERK was not involved in the up-regulation of VCAM-1 by HLA I Abs in our experimental system of primary human ECs. Again, these contradictory observations may be explained by cell type-specific differences in various cell culture models. It is also interesting to note that in contrast to the NF-κB-regulated genes VCAM-1, ICAM-1, IL-8 and MCP-1, expression of the NF-κB-dependent gene Cox-2 [39] was not affected by HLA I Abs (Fig 1B). This may suggest that additional regulatory factors and pathways are involved in HLA I Ab-dependent EC activation. Remarkably, complement-derived membrane attack complexes have recently been reported to markedly enhance HLA Ab-dependent induction of proinflammatory genes in ECs without causing cytolysis of these cells [53]. Thus it is conceivable, that complement also plays a key role in HLA Ab-dependent EC activation, the mechanisms of which still remain to be explored in further detail.

### HO-1 modulates HLA I Ab-dependent EC activation

Our current findings show that HLA I Ab-mediated activation of human ECs is modulated by HO-1 (Fig 3). The data correspond with previous reports on mouse and human genetic HO-1 deficiency, which revealed endothelial protective properties against prooxidant and proinflammatory damage for this enzyme [21, 22]. Independently, overexpression of HO-1 has been shown to inhibit TNF-α-mediated induction of proinflammatory inducible adhesion molecules in cell cultures of primary bovine ECs [23]. As this HO-1 effect has been ascribed to inhibit NF-κB [40], such mechanism might also be involved in HO-1-dependent inhibition of HLA I Ab-dependent EC activation in our cell culture system (Fig 3). Remarkably, HO-1 induction has also been demonstrated to counteract the pro-apoptotic effects of Abs against the Dengue virus non-structural protein 1 [34] and complement-dependent effects of HLA I Abs in the endothelium [30]. CO, which is one of the products of HO, is known to have major therapeutic potential in various physiological and pathophysiological settings [42]. As demonstrated in Fig 4, CO mediated HO-1-dependent modulation of the HLA class I Ab-mediated induction of VCAM-1 gene expression in HUVECs (Fig 4). This latter observation supports earlier studies showing that HO-1-derived CO protects against the rejection of mouse to rat cardiac transplants [28, 41].

### HO-1 as a therapeutic target for treatment of AMR

Activation of ECs is critical for the pathogenesis of various inflammatory vascular disorders including AMR [14, 54]. Proinflammatory alterations of graft arteries are a characteristic feature of AMR, which has been demonstrated in animal models [55, 56]. The coordinate up-regulation of inducible surface proteins such as selectins and adhesion molecules on ECs is essential for leukocyte recruitment to the site of an inflammation [16, 17]. Our functional studies in a monocyte adhesion assay also confirmed that EC activation by HLA I Abs mediates binding of THP-1 monocytes to ECs (Fig 5), which corresponds to previous findings by others [36, 44]. Remarkably, Valenzuela and colleagues have recently demonstrated a major role for P-selectin in HLA I Ab-mediated recruitment of monocytes to ECs [44]. The clinical success rate of current therapeutic regimens in AMR including treatment with CD20 Abs (rituximab) and/or plasmapheresis, both of which primarily aim to reduce levels of circulating DSAs in the serum [4, 18, 19], is poor and novel complementary therapy strategies are urgently needed. Thus, specific up-regulation of anti-inflammatory protective mechanisms in the endothelium could be an alternative therapeutic approach in AMR. For example, a recombinant designer protein, which binds to activated endothelium via an E-selectin-interacting domain, has recently been shown to block activation of ECs via inhibition of NF-κB [57]. Similarly, targeted pharmacological up-regulation of endothelial HO-1 might counteract AMR-associated inflammation in heart and kidney transplantation. A potential pharmacological strategy to achieve this goal, could be the application of statins, which have previously been shown to up-regulate HO-1 in the endothelium [58–60]. Statins are widely used for the treatment of cardiovascular diseases and might also be applicable for AMR therapy.

In conclusion, we have shown that HLA I Ab-dependent activation of human ECs is counteracted by up-regulation of the anti-inflammatory enzyme HO-1. Therefore, targeting of endothelial HO-1 might be a promising strategy for the treatment of AMR in solid organ transplantation.
